# Comparison of Type 2 Diabetes Mellitus Control at Home Healthcare and Hospital Clinic Care at King Salman Armed Forces Hospital (2021-2022): A Retrospective Cohort Study

**DOI:** 10.7759/cureus.48551

**Published:** 2023-11-09

**Authors:** Rofayda Mansour Ahmed Mohamad, Salem Khalil Adhahi, Manea Nasser Alhablany, Hiba Mustafa Abdelrahman Hussein, Tayseer Mubarak Eltayb, Samir Salah Eldin Mohamed Buraei, Abdulaziz Awadh Alshamrani, Manar Suliman Manqarah, Dalal Eid Alhowiti, Abrar Mubarak Aloqbi, Kholoud Ali Salamah Alatawi, Rahaf Mubarak Aloqbi

**Affiliations:** 1 Preventive Medicine Department, King Salman Armed Forces Hospital, Tabuk, SAU; 2 Home Health Care Department, King Salman Armed Forces Hospital, Tabuk, SAU; 3 Chronic Illness Control Department, King Salman Armed Forces Hospital, Tabuk, SAU; 4 Family Medicine Department, King Salman Armed Forces Hospital, Tabuk, SAU; 5 Paediatric Intensive Care Unit, King Salman Armed Forces Hospital, Tabuk, SAU

**Keywords:** type 2 diabetes mellitus, glycemic control, glycosylated hemoglobin, hospital clinic care, home healthcare

## Abstract

Background: Home healthcare represents a great necessity for patients with diabetes mellitus (DM). Although there are numerous studies on geriatric diabetic patients, there are few studies on diabetic home care versus hospital care.

Aim: This study aimed to compare the effect of home healthcare services to hospital care for controlling type 2 diabetes mellitus (T2DM) at King Salman Armed Forces Hospital, Tabuk, Saudi Arabia.

Methods: This retrospective cohort study included patients with type 2 diabetes mellitus at King Salman Armed Forces Hospital. The home healthcare group included 128 participants who received frequent follow-up visits at home. The hospital care group included 128 participants from the primary care clinic. Glycosylated hemoglobin (HbA1c) was used to measure glycemic control. Logistic regression analysis was done to detect factors related to achieving glycemic control.

Results: Home healthcare care had a greater impact on the reduction of baseline glycosylated hemoglobin levels (p=0.0053). The target glycosylated hemoglobin was achieved by patients who received home healthcare (p=0.020). Using the multivariate regression analysis, home healthcare, married patients, those who can do full daily life activities without assistance, and those who were treated with only insulin had significant correlations to meet the target glycosylated hemoglobin level (odds ratio: 0.814, 0.541, 0.448, and 0.144; 95% confidence intervals: 0.72-0.94, 0.30-0.99, 0.31-0.65, and 0.08-0.25, respectively).

Conclusions: Home care strategy for patients suffering from type 2 diabetes mellitus provides better glycemic control compared to hospital care. Home care, marriage, doing full daily activities, and insulin treatment are important factors affecting glycemic control.

## Introduction

Diabetes mellitus (DM) is a common chronic health issue. Saudi Arabia is one of the top 10 nations with the highest prevalence of diabetes. By 2045, it is anticipated that Saudi Arabia will rank among the top 5 countries with the highest prevalence of type 2 diabetes mellitus (T2DM) [1].

Numerous risk factors for developing diabetes mellitus have been found among Saudi adults, including eating habits, inactivity, obesity, and urbanization [2]. Saudi patients have low knowledge of T2DM risk factors and preventive interventions and a high incidence of physical inactivity [3,4].

National and international guidelines recommend routine measurements for capillary blood glucose, blood pressure, and body mass index [5]. People with diabetes must learn to manage their condition daily [6]. For older adults with diabetes, evidence-based guidelines recommend a glycosylated hemoglobin (HbA1c) level between 7% and 8.5%. If these recommendations are followed, diabetes-related adverse events may be reduced [7].

Unfortunately, most patients with T2DM do not meet the recommended treatment targets. Hence, major complications might develop, affecting the heart, eyes, kidneys, and feet [8]. In comparison to people without diabetes, adults over 50 with diabetes have a reduced life expectancy of 7.5-8.2 years [9].

Moreover, elderly people frequently have comorbid conditions, which are linked to rapid health reductions and a higher risk of impairment. High rates of comorbidity can make it difficult to care for the elderly [10]. In addition, mortality rates tend to be elevated in older people with diabetes due to an augmented susceptibility to depression. Consequently, it is necessary to implement regular and meticulous screening and treatment procedures to evaluate these individuals' medical, psychological, functional, and social aspects. The rise in life expectancy and technological advancements have led to a significant surge in the elderly population receiving home care services [11]. These services contribute to the development of effective therapeutic strategies and the establishment of glycemic control objectives. Hence, home healthcare plays a crucial source in effective medical treatment for the elderly population [11].

Pre-hospital care may represent an alternative to hospital services. In recent years, there has been an increase in the demand for home healthcare. More people are turning to home health services to provide patients with mobility issues with services in a pleasant setting, safeguard the patient from hospital infections, and reduce the need for unnecessarily lengthy hospital stays. Furthermore, home services may be able to avoid the high capital expenses involved in constructing and maintaining major hospitals [11,12].

In light of the increasing demand for and significance of home health services, it is imperative to enhance our understanding of strategies to mitigate or prevent issues and inaccuracies associated with the receipt of home healthcare. Mortality related to medical errors is increasing globally. The estimations provided are derived from research conducted in hospital settings; however, it is probable that a significant proportion of individuals also experience mortality due to errors occurring within home healthcare settings. Human factor interventions possess the capacity to mitigate these errors and consequently preserve human lives [13].

Studies comparing diabetic patients receiving home care to those receiving hospital healthcare services are limited. Therefore, this study was conducted to explore the outcome of home healthcare compared to hospital clinic care for controlling T2DM at King Salman Armed Forces Hospital, Tabuk, Saudi Arabia.

## Materials and methods

Ethical considerations

The protocol of this study was approved by the Research Ethics Committee of King Salman Armed Forces Hospital, Tabuk, Saudi Arabia (ID: KSAFH-REC-2022-443). Before the data collection process, all participants were informed about the study objectives and methodology, and informed consent was obtained from each one. The participants' data were kept confidential.

Study design and setting

This retrospective cohort study was conducted at King Salman Armed Forces Hospital, Tabuk, Saudi Arabia, between January 2023 and July 2023.

Eligibility criteria

The study enrolled type 2 diabetic patients aged 18 years or more on regular follow-up either by home healthcare or primary care clinics at King Salman Armed Forces Hospital. Type 1 diabetic patients and those not treated at the hospital were excluded.

Data collection tool

This study included 256 participants who were diagnosed with T2DM. The hospital care group included 128 participants who received routine care, including inpatient hospital services and monthly visits to the diabetes clinic. The home healthcare group included 128 participants who received monthly follow-up nurse visits and consultations at home, with regular monitoring of their HbA1c level (every three months). If the glucose level was uncontrolled or there was a change in the treatment, the visits were scheduled to be more frequent (up to a weekly visit) till good control of the glucose level was achieved. In addition, the communication with the patient was available through a hotline. Age, sex, education, body built, time of diagnosis, and socioeconomic status were well-matched in both groups. The follow-up visits were designed to be for at least three years.

Data gathering was performed using a questionnaire to record the patients' characteristics. Demographic, constitutional, socioeconomic, and follow-up control measures including adherence to medication, activity, mobility, nutrition, comorbidities, and blood glucose were utilized to evaluate the performance of the two employed approaches. Glycosylated hemoglobin measured the risk of long-term consequences from diabetes mellitus as well as overall glycemic exposure [14]. The American Geriatrics Society proposed an overall HbA1c target of 7.5%-8% as the therapeutic objective for glycemic management in elderly adults (>65 years) [15].

The questionnaire was revised after a pilot study to make sure that all questions were precise and easy to comprehend. To guarantee that the survey was conducted consistently, all the investigators obtained standardized training. The questionnaire was completed by the participants on their own with the support and assistance of an on-site investigator. The investigators immediately verified and gathered completed surveys.

Sample size

According to Albarakat and Guzu [10], the total sample size was estimated to be 256 participants, assuming that the odds ratio for the outcome in both groups was 4. The sample size was calculated with the statistical software Population Proportion Sample Size according to the following formula: sample size (n) = N*X/(X + N‑1), where X = Za/2, 2*p*(1‑p)/MOE2. Za/2 is the critical value of the normal distribution for a confidence level of 95%, P is the migration error, P is the sample proportion, and N is the population size.

Statistical analysis

Tabulation and analysis of data were carried out using the Statistical Package for the Social Sciences software (SPSS) version 26 (SSPS, Inc., Chicago, IL). Categorical variables were presented as numbers and percentages and were compared using Pearson's chi-square (χ2) test, Fisher's exact test, or Cochran-Armitage test as appropriate. Continuous variables were expressed as the mean ± standard deviation (SD) and were compared using the Mann-Whitney test. Multivariate logistic regression analysis was used to assess the association of different variables with the HbA1c level. A p-value < 0.05 was considered statistically significant.

## Results

Married patients represent a significantly high percentage of the home healthcare group (p<0.001), whereas unemployed, non-smoker patients, with 1-2 comorbidities who had experienced T2DM for more than 10 years were significantly higher in the hospital clinic care group (p<0.001, 0.006, <0.001, and 0.002, respectively) (Table 1).

**Table 1:** Demographic, Clinical, and Diabetic Characteristics of the Study Participants Data are presented as number of patients and percentage. *Significant at p<0.05

		Hospital clinic care		Home healthcare		p-value
		n=128	%	n=128	%	
Age (years)	49 and below	7	5.5	7	5.5	1.00
	50-60	9	7	9	7	
	61-70	35	27.3	35	27.3	
	71-80	53	41.4	53	41.4	
	81-90	21	16.4	21	16.4	
	91 and above	3	2.3	3	2.3	
Gender	Female	85	66.4	85	66.4	1.00
	Male	43	33.6	43	33.6	
Occupation	Employed	4	3.1	2	1.6	<0.001*
	Unemployed	114	89.1	61	47.7	
	Dependent	10	7.8	65	50.8	
Education	Illiterate	70	54.7	72	56.3	0.677
	Primary	35	27.3	36	28.1	
	Secondary	11	8.6	13	10.2	
	Tertiary	12	9.4	7	5.5	
Marital status	Single	0	0	3	2.3	<0.001*
	Married	69	53.9	96	75	
	Divorce/separated	6	4.7	4	3.1	
	Widowed	53	41.4	25	19.5	
Body built	Underweight	2	1.6	3	2.3	0.065
	Average	48	37.5	56	43.8	
	Overweight	68	53.1	49	38.3	
	Obese	10	7.8	20	15.6	
Smoking	Smoker	7	5.5	21	16.4	0.006*
	Non-smoker	121	94.5	107	83.6	
Comorbidities	None	2	1.6	3	2.3	<0.001*
	1-2 diseases	111	86.7	43	33.6	
	3 diseases	15	11.7	82	64.1	
Duration of diabetes	0-5 years	14	10.9	10	7.8	0.002*
	5-10 years	18	14.1	42	32.8	
	More than 10 years	96	75	76	59.4	
Time of diagnosis	Early diagnosed (before 5 years)	117	91.4	120	93.8	0.474
	Recently diagnosed (within 5 years)	11	8.6	8	6.3	

The highest percentage of patients under home healthcare need assistance to do activities of daily living (ADLs) or can do it partially, and patients in the hospital clinic care were followed up for more than 10 years. Fully oriented, independent diabetic patients were significantly managed in the hospital care clinic compared to the home care (p<0.001). High percentages of patients in hospital healthcare were adherent to medication, although they were not adherent to the diet plan compared to those under home care. Oral antidiabetic drugs only or both oral drugs and insulin were the commonest type of medications among patients under hospital care. Patients who received hospital care had no limitation to mobility, could independently feed orally with adequate nutritional status, and did not need a supplemented formula with a statistically higher percentage than those who received home healthcare (p<0.001) (Table 2).

**Table 2:** Relevant Characteristics, Management Plan, and Adherence of the Study Participants ADLs: activities of daily living *Significant at p<0.05

		Hospital clinic care		Home healthcare		p-value
		n=128	%	n=128	%	
Functional assessment	Can do ADLs fully	74	57.8	8	6.3	<0.001*
	Can do ADLs partially/with assistance	46	35.9	88	68.8	
	Dependent	8	6.3	32	25	
Length of follow-up	Less than 1 month	1	0.8	1	0.8	<0.001*
	1-2 months	0	0	12	9.4	
	3-6 months	2	1.6	6	4.7	
	7 months-1 year	2	1.6	4	3.1	
	2-3 years	6	4.7	26	20.3	
	4-7 years	17	13.3	55	43	
	8-10 years	10	7.8	12	9.4	
	More than 10 years	90	70.3	12	9.4	
Cognitive function	Well-oriented	116	90.6	78	60.9	<0.001*
	Not fully oriented	6	4.7	37	28.9	
	Not oriented/demented	6	4.7	13	10.2	
Caregiver	Wife	0	0	13	10.2	<0.001*
	Husband	0	0	3	2.3	
	Daughter	9	7	33	25.8	
	Son	9	7	27	21.1	
	Home nurse	0	0	2	1.6	
	Caregiver	27	21.1	36	28.1	
	Independent	83	64.8	14	10.9	
Adherence to medication	Yes	85	66.4	64	50	0.029*
	Sometimes (on and off)	37	28.9	55	43	
	No	6	4.7	9	7	
Adherence to the diet plan	Yes	23	18	42	32.8	0.014*
	Sometimes (on and off)	87	68	76	59.4	
	No	18	14.1	10	7.8	
Types of medications	No medication	2	1.6	6	4.7	<0.001*
	Oral antidiabetic drugs only	64	50	49	38.3	
	Insulin only	4	3.1	29	22.7	
	Both oral and insulin	58	45.3	44	34.4	
Activity	Bedfast	3	2.3	23	18	<0.001*
	Chair fast	13	10.2	45	35.2	
	Walks frequently	5	3.9	14	10.9	
	Walks occasionally with assistance	31	24.2	44	34.4	
	No limitation to mobility	76	59.4	2	1.6	
Source of nutrition	Oral feeding independently	122	95.3	78	60.9	<0.001*
	Oral feeding with assistance	4	3.1	46	35.9	
	Nasogastric tube	1	0.8	4	3.1	
	Percutaneous endoscopic gastrostomy tube	1	0.8	0	0	
Nutritional status	Adequate	110	85.9	84	65.6	<0.001*
	Probably inadequate	12	9.4	44	34.4	
	Very poor	6	4.7	0	0	
On supplemented formula	Yes	5	3.9	26	20.3	<0.001*
	No	123	96.1	102	79.7	

High percentages of patients under home healthcare had reduced HbA1c from the baseline value (p=0.0053). Moreover, they had experienced meeting the target HbA1c compared to patients under hospital clinic care (p=0.020). Glucose monitoring was significantly higher among patients under home care than those under hospital care. Meanwhile, blood pressure and caregiving were well-controlled in patients under hospital care than those under in-home care (p<0.001). Better outcomes including mood disorder, kidney function, diabetic foot, and other presented wounds or heart diseases were markedly noted in patients under hospital clinic care compared to those under home healthcare (p<0.001) (Table 3).

**Table 3:** Diabetes Control, Meeting the Target HbA1c, Follow-Up, Caregiving, and the Outcome of the Study Participants HbA1c: glycosylated hemoglobin, CI: confidence interval, OR: odds ratio, SD: standard deviation, DM: diabetes mellitus *Significant at p<0.05

		Hospital clinic care (n=128)		Home healthcare (n=128)		
Diabetes control (mean±SD)	Baseline HbA1c	8.8±1.9		8.3±2.0		
	Latest HbA1c	8.4±1		7.6±1.9		
	p-value	0.0886		0.0053*		
		Number	%	Number	%	p-value
Target HbA1c	Met	60	46.9	78	60.9	0.020*
	Not met	68	53.1	50	39.1	
Glucose monitoring is done by the patient and/or caregiver	Yes	54	42.2	91	71.1	<0.001*
	Sometimes	54	42.2	0	0	
	No	20	15.6	37	28.9	
Blood pressure	Controlled	120	93.8	82	64.1	<0.001*
	Uncontrolled	8	6.3	46	35.9	
Caregiving	Well cared	103	80.5	55	43	<0.001*
	Average	25	19.5	69	53.9	
	Neglected	0	0	4	3.1	
Mood disorder	None	127	99.2	60	46.9	<0.001*
	Stress	1	0.8	26	20.3	
	Depression on treatment	0	0	6	4.7	
	Depression not on treatment	0	0	32	25	
	Not oriented	0	0	4	3.1	
Renal complication	Normal kidney function	118	92.2	46	35.9	<0.001*
	Chronic kidney disease 1-2	9	7	56	43.8	
	Chronic kidney disease 3	1	0.8	11	8.6	
	Chronic kidney disease 4-5	0	0	10	7.8	
	End-stage renal disease	0	0	5	3.9	
Diabetic foot	No history	124	96.9	110	85.9	<0.001*
	Post-amputation of toes or limb	0	0	9	7	
	With DM foot now	4	3.1	0	0	
	With DM foot history	0	0	9	7	
Other wounds presented	Pressure ulcer	2	1.6	2	1.6	<0.001*
	Leg ulcers	2	1.6	1	0.8	
	Postoperative wounds	5	3.9	1	0.8	
	With a history of wounds healed recently	2	1.6	34	26.6	
	None	117	91.4	90	70.3	
Heart disease	Ischemic heart disease	6	4.7	29	22.7	<0.001*
	Congestive heart failure	1	0.8	2	1.6	
	Atrial fibrillation	2	1.6	5	3.9	
	Others	119	93	92	71.9	

Multivariate logistic regression analysis was done for the detection of the relationship between factors in T2DM patients to meet the target HbA1c. Patients who received home healthcare services, married patients, patients who could do ADLs fully, and those treated with insulin only significantly met the target HbA1c (odds ratio: 0.814, 0.541, 0.448, and 0.144, with confidence intervals 0.72-0.94, 0.30-0.99, 0.31-0.65, and 0.08-0.25, respectively) (Table 4).

**Table 4:** Multivariate (Logistic Regression) Analysis for the Relationship Between Factors Meeting the Target HbA1c HbA1c: glycosylated hemoglobin, ADLs: activities of daily living, CI: confidence interval, OR: odds ratio, BMI: body mass index, DM: diabetes mellitus *Significant at p<0.05

Predictors	Odds ratio	p-value	95% CI (from-to)	
Place of management (home healthcare)	0.814	0.020*	0.72	0.94
Age	1.215	0.649	0.52	2.81
Gender	1.330	0.171	0.88	2.00
Occupation	1.220	0.678	0.48	3.12
Marital status (married)	0.541	0.047*	0.30	0.99
BMI	0.920	0.508	0.72	1.18
Smoking	1.938	0.089	0.91	4.15
DM duration	0.932	0.532	0.75	1.16
Time of diagnosis	1.088	0.697	0.71	1.66
Function status (full ADLs)	0.448	<0.001*	0.31	0.65
Follow-up length	0.767	0.134	0.54	1.08
Cognitive function	0.636	0.303	0.27	1.50
Caregiver identity	0.722	0.427	0.32	1.61
Medical adherence	0.414	0.214	0.10	1.66
Diet adherence	0.764	0.484	0.36	1.62
Medical type (insulin only)	0.144	<0.001*	0.08	0.25
Activity	0.778	0.578	0.32	1.89
Nutrition source	1.229	0.575	0.60	2.53
Nutrition status	1.061	0.717	0.77	1.46
Supplement	1.063	0.785	0.68	1.65
Comorbidities	0.812	0.504	0.44	1.50

## Discussion

Type 2 diabetes mellitus is a chronic health condition. Maintaining excellent metabolic control may help postpone its complications [16]. Governments and health institutes must raise the standard of healthcare. Home-based care provides great benefits in managing chronic illnesses [17,18]. There are a lot of studies on treating elder diabetic patients in the literature, but not enough studies on diabetic patients receiving home healthcare compared to hospital care clinics [5,11,12]. Thus, this study aimed to explore the effect of home healthcare services compared to hospital clinic care for controlling T2DM at King Salman Armed Forces Hospital, Tabuk, Saudi Arabia.

Our main findings revealed that home-based care had a greater impact on controlling glucose levels that met the target HbA1c level. By multivariate logistic regression analysis, home healthcare, marital status, the ability to do ADLs fully without any assistance, and treatment with insulin only were significant factors affecting patients to meet the target HbA1c level.

Demographic, clinical, diabetic, and other relevant characteristics of patients in home healthcare were in line with previous studies [10,11,18,19], where married, dependent females who had more than three diseases received home healthcare more frequently. Meanwhile, independent, fully oriented patients who can do their daily activities without any assistance and were diagnosed with T2DM for more than 10 years significantly received treatment at hospital healthcare services. A recent meta-analysis [20] found that the incidence and prevalence rates of DMT2 were rising among females and in urban areas of the Eastern Province, Jeddah, and Riyadh more rapidly than in rural areas. This was important to comprehend the needs and characteristics of patients receiving home healthcare to ensure that the providers' healthcare support services are as effective as possible [21].

The presence of a caregiver, a comprehensive understanding of the causes and risk factors associated with T2DM, and self-care practices, such as adhering to medication regimens, maintaining a healthy diet, regularly monitoring blood sugar levels, and engaging in proper foot care, were essential in effectively managing T2DM. Home healthcare services can enhance glycemic control and mitigate the development of related comorbidities. Similarly, Albarakat and Guzu [10] reported that providing home healthcare services is crucial in managing and preventing diabetes and its associated complications among elderly individuals. Moreover, the correlation between home healthcare and engagement in self-care practices exhibits encouraging prospects. Enhanced patient-provider connections, along with heightened patient education and comprehension of diabetes, support from healthcare professionals and social networks, and engagement facilitated by healthcare are anticipated to promote greater awareness and adherence to self-care practices.

Home healthcare was frequently used to manage patients with mobility issues who could not do daily activities and needed assistance for nutritional support. Consequently, their adherence to treatment and diet plans was restricted [12,13]. Istek and Karakurt [22] reported that individuals with diabetes should schedule routine checkups, monitor their blood sugar levels, follow insulin and medication regimens, and plan their diet and exercise regimens to achieve glycemic control.

Nutritional state and adherence to a diet plan are important confounders. Bulucu-Büyüksoy and Karataş [12] demonstrated that patients had challenges in effecting behavioral changes, particularly in exercise. Furthermore, patients quickly abandoned their acquired knowledge and reverted to their previous behaviors. Patients' ability to modify their eating habits was hindered by factors such as lack of knowledge, economic constraints, resistance to altering food culture, and social norms associated with public dining occasions.

The target glycemic control should be monitored according to the patient's comorbidities and cognitive functions. Patients with multiple comorbidities and cognitive impairment were advised to have fewer strict glycemic goals as HbA1c of 8%-8.5%. Meanwhile, older adults with few coexisting comorbidities and intact cognitive function were advised to have lower glycemic goals as HbA1c of 7.5% [23]. Elderly diabetic individuals with HbA1c levels ranging from 7% to 8% had the best survival rates [24,25].

In the current study, patients benefit from home healthcare, which lowered HbA1c levels from 8.3% to 7.6% and frequently met the target compared to patients who received hospital healthcare. Janati et al. [19] found a mean of 37% reduction in HbA1c levels among patients receiving home-based care for three months. Bulucu-Büyüksoy and Karataş [12] reported a significant reduction of HbA1c by 0.75% in patients with T2DM who received home care. Albarakat and Guzu [10] found that the majority of cases had satisfactory glycemic control among home-cared patients at Al Kharj Military Industries Corporation Hospital, Saudi Arabia. Nearly 60% of bedridden patients and 70% of those utilizing chair wheels achieved their desired glycemic targets. Armour et al. [26] documented that family interventions among relatives or housemates of people with diabetes were successful in enhancing the understanding of diabetes and glycemic management. Moreover, Jafary et al. [18] demonstrated that providing home healthcare to patients with diabetic foot ulcers was more cost-effective than providing hospital care. The frequency of blood glucose monitoring for patients under home healthcare had increased. It could be attributed to the fact that most participants and/or their caregivers acquired the skills to measure blood glucose and formed a routine to do so. The strength of our findings was that the substantial reduction of HbA1c level in in-home care was demonstrated by the fact that the mean HbA1c level at baseline was practically comparable (8.8±1.9 versus 8.3±2.0 mg/dL) between both hospital and home care groups.

Caregivers significantly affect home healthcare services. The involvement of patients' family members serves as an external source of motivation for engaging in blood glucose management. Enhancing family support and enhancing knowledge and attitudes toward diabetes are expected to have a positive impact on patients' health behaviors and overall outcomes [27]. Additionally, Linekin [28] declared that home healthcare offers emotional and physical stress alleviation for sufferers within the comfort of their residences, especially for diabetic patients. The majority of stresses have the potential to elicit elevations in counter-regulatory hormones. This phenomenon can worsen insulin resistance, triggering the release of glucose from the liver and consequently causing an increase in blood glucose levels. Providing therapeutic support and attentive care by home healthcare has the potential to mitigate the physiological and psychological stress responses experienced by elders. Furthermore, home healthcare providers can mitigate external environmental stresses by facilitating access to supplementary services, such as those rendered by a home health aide, physical therapist, social worker, or community program (e.g., Meals on Wheels), as deemed necessary.

In contrast, Johnson et al. [29] demonstrated that community health workers' programs failed to produce the anticipated results due to poor training, competence, and systemic support. Lack of care coordination and provider clinical inertia (i.e., slowness to appropriately enhance diabetic therapy) are two aspects of hospital healthcare delivery that significantly contribute to the poor metabolic control seen in T2DM [30]. In addition, the presence of patients within general hospitals results in the occurrence of overcrowding, extended waiting periods, and excessive strain on transportation and medical resources [31].

Marital status could affect the patient's response to meet the target HbA1c. Some couples may eat more and be less active, which might increase body weight and disease risk [32]. Furthermore, marriage has an impact on a person's lifestyle. Widowed, divorced, and/or separated patients prefer to be independent, and they prefer to get help without assistance at the hospital clinic [13].

Patients who can do full daily activities without any assistance were significantly able to meet the target reduction of HbA1c. Jie et al. [33] noted that the prevalence of functional restrictions among older persons (>70 years) with T2DM was high across a variety of daily living activities, which negatively impacts each person's quality of life, their families, the healthcare system, and the entire community.

Type 2 diabetes mellitus is brought on by hyperglycemia from insulin resistance or inadequate insulin production from the pancreatic beta cells [34]. The type of medications had a great impact on the target HbA1c. In this study, greater insulin use could be attributed to older age, greater comorbidities, and a longer duration of diabetes of more than 10 years. Heimro et al. [5] and Sertbas et al. [11] reported that a considerably larger percentage of insulin users in their study had HbA1c readings within the recommended range in comparison to those not using insulin.

The reduction of HbA1c could result in a reduction of the subsequent complications. Each 1% reduction in HbA1c lowered the risk of myocardial infarction by 14%, diabetes-related mortality by 21%, and microvascular complications by 37%, as well as the chance of developing eye, kidney, and nerve illness by 40% [35,36]. Home healthcare services are important for diabetic geriatric patients. Healthcare policymakers should develop a variety of home healthcare programs to educate patients about potential risk factors and diabetes comorbidities.

Limitations

This retrospective cohort study should have been extended to meet the time needed for the target reduction of HbA1c. Hypoglycemic conditions were not demonstrated as important adverse events during the control of diabetes mellitus. The self-administered questionnaires were subjected to the possibility of recall bias. This research was carried out at King Salman Armed Forces Hospital; hence, the findings may not represent the broader community in Saudi Arabia. However, our results may pave the way for larger, multicenter studies including most, if not all, of the Saudi hospitals.

## Conclusions

Home healthcare provides better control for glycosylated hemoglobin compared to hospital healthcare. The place of management, marital status, functional status, and type of medication have significant impacts on achieving the target glycemic control.
